# Characterization and Pharmacokinetic Study of Aprepitant Solid Dispersions with Soluplus^®^

**DOI:** 10.3390/molecules200611345

**Published:** 2015-06-19

**Authors:** Jinwen Liu, Meijuan Zou, Hongyu Piao, Yi Liu, Bo Tang, Ying Gao, Ning Ma, Gang Cheng

**Affiliations:** Department of Pharmacy, Shenyang Pharmaceutical University, No. 103 Wenhua Road, Shenyang 110016, China; E-Mails: liujinwen7@sina.com (J.L.); zoumeijuan@163.com (M.Z.); HY_piao@126.com (H.P.); woshi_yybb@163.com (Y.L.); syputony@163.com (B.T.); gaoy1900@163.com (Y.G.); mn1989110500@163.com (N.M.)

**Keywords:** aprepitant, Soluplus^®^, solid dispersions

## Abstract

Solid dispersions are a useful approach to improve the dissolution rate and bioavailability of poorly water-soluble active pharmaceutical ingredients (APIs). The aim of this study was to improve the physicochemical properties and bioavailability of a poorly water-soluble aprepitant by preparation of solid dispersions. The solid dispersions were characterized by dissolution, FTIR, XRPD, DSC, SEM and pharmacokinetic studies in rats. The dissolution rate of the aprepitant was significantly increased by solid dispersions, and XRD, DSC, and SEM analysis indicated that the aprepitant existed in an amorphous form within the solid dispersions. The result of dissolution study showed that the dissolution rate of SDs was nearly five-fold faster than aprepitant. FTIR spectrometry suggested the presence of intermolecular hydrogen bonds between the aprepitant and polymer. Pharmacokinetic studies in rats indicated that the degree drug absorption was comparable with that of Emend^®^. Aprepitant exists in an amorphous state in solid dispersions and the solid dispersions can markedly improve the dissolution and oral bioavailability of the aprepitant. The AUC_0–t_ of the SDs was 2.4-fold that of the aprepitant. In addition, the method and its associated techniques are very easy to carry out.

## 1. Introduction

Substance P is the most abundant neurokinin in the mammalian CNS and a potent modulator of neuroimmunoregulation [1,2]. Aprepitant is a selective high affinity antagonist of human substance P/neurokinin 1 (NK1) receptors and has little or no affinity for serotonin (5-HT3), dopamine and corticosteroid receptors, which are the targets of existing therapies for chemotherapy-induced nausea and vomiting as well as postoperative nausea and vomiting [3,4,5]. Aprepitant is a basic compound with a pKa value of 9.7 within the pH range 2 to 12. The free base aqueous solubility (3–7 μg/mL) is very low over the pH range 2–10. The compound has a logP value of 4.8 at pH 7.0 [6,7]. Because of its poor solubility, it is difficult to develop a formulation using conventional approaches that will provide adequate systemic exposure to produce a therapeutic effect [8]. The currently marketed formulation of aprepitant (Emend^®^) is based on nanoparticle technology in which its solubility is increased using drug nanoparticles. The complex nature of nanoparticle technology in terms of processing and effort required necessitate exploration of alternate technologies for solubility enhancement [9,10].

Several techniques can be successfully used to improve the dissolution and bioavailability of poorly water soluble drugs, use of surfactants, particle size reduction [11,12], salt formation, cyclodextrin inclusion complexation [13,14], solid dispersions [15,16], pH adjustment, and the use of pro-drugs or incorporation of the drug in polymeric or lipid formulations [17]. The majority of these approaches have been used on a case by case basis depending on the physicochemical characteristics of the drug. Among all the above mentioned approaches, solid dispersions seems to be a simple approach for developing commercially viable dosage forms of poorly soluble compounds. The method of solvent evaporation involves the solubilization of the drug and polymer in a solvent or a mixture of solvents, which is then evaporated [18]. This method is very simple, convenient and good reproducibility. Although solvent evaporation method has been extensively confirmed to enhance the dissolution characteristics of the sparingly soluble drugs, the practical applicability of the system has remained limited mainly due to the difficulties in manufacturing process, such as the complexity of dosage form development and less feasibility for scale up of industrialization [19]. Soluplus^®^ (polyvinyl caprolactam–polyvinyl acetate–polyethylene glycol graft copolymers), a new polymer with amphiphilic properties was used [20]. It is recommended for solubilizing poorly soluble APIs due to the amphiphilic nature of the polymer [21].

Thus, the present study was undertaken to prepare SDs of aprepitant that would offer a maximum increase in dissolution and oral bioavailability using a minimal amount of carrier. The prepared SDs were characterized by their saturation dissolution, FTIR, PXRD, DSC, SEM, stability and pharmacokinetics in rats.

## 2. Results and Discussion

### 2.1. Dissolution Studies

**Figure 1:**
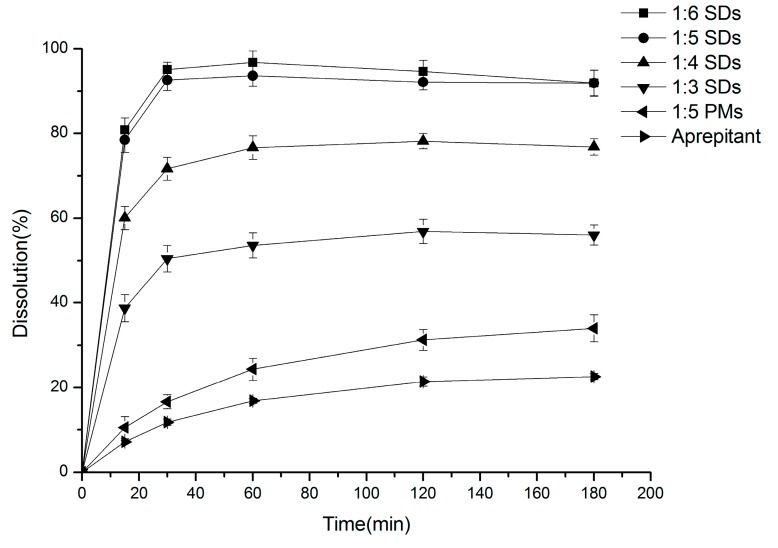
Dissolution profile of aprepitant and solid dispersions at different aprepitant/Soluplus^®^ ratios of 1:6, 1:5, 1:4, 1:3 and PMs. Each point represents the mean ± SD (*n* = 3).

The release rates of the different SDs of aprepitant in PBS pH 6.6 containing 0.1% SDS and the pure drug are compared in Figure 1.

As can be seen, all the SDs were able to increase the dissolution rate of aprepitant. These data are in agreement with the increase in drug solubility in these formulations. Pure aprepitant exhibited a low dissolution rate, with a 11.77% ± 0.77% release in 30 min and only reached 22.61% ± 0.74% after 180 min. Physical mixtures released 33.98% ± 3.16% of drug at 180 min. Furthermore, as we expected, an apparent trend was observed between the Soluplus^®^ ratios and the dissolution rate of aprepitant. The formulation 1:6 SDs released 95.07% ± 1.84% of drug in 30 min, which indicated a 9-fold increase in drug dissolution during this period. The formulations with 1:5, 1:4 and 1:3 SDs exhibited a release of 92.67% ± 2.55%, 71.7% ± 3.98% and 50.43% ± 3.11%, respectively. The formulations with 1:6 and 1:5 SDs showed similar release curves and higher dissolution rates than the 1:4 and 1:3 SDs formulations. Therefore, the formulation 1:5 SDs were chosen for further study.

Faster dissolution of aprepitant in solid dispersions might be explained by improved drug wetting in the dissolution medium and the conversion from the drug crystalline to the amorphous state (as indicated by XRD and DSC results) [22]. The dissolution rate of the resultant formulation was greatly affected by the physical state of the dispersed drug. The amorphous state tends to be more soluble since no energy is required to break up the crystal lattice during the dissolution process [23].

### 2.2. Differential Scanning Calorimetry (DSC)

**Figure 2:**
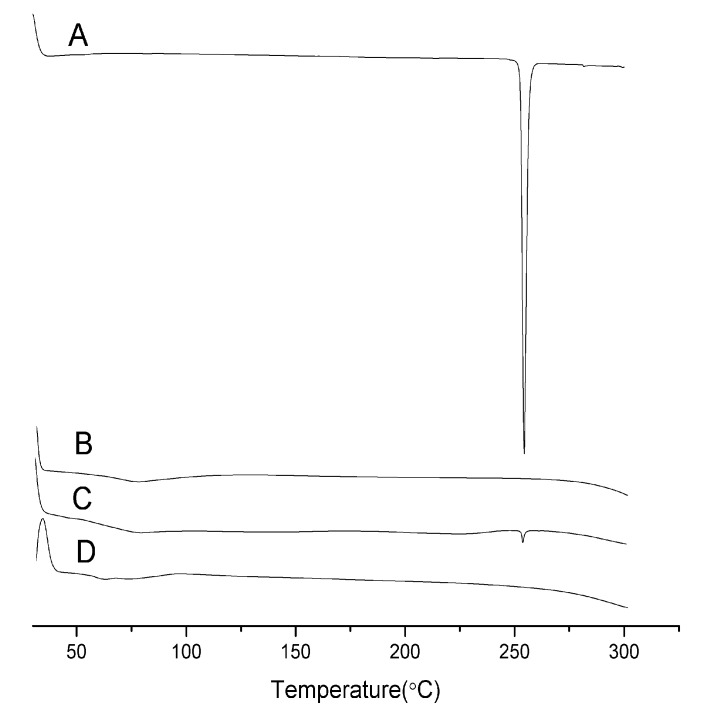
DSC curves of Aprepitant (**A**); Soluplus^®^ (**B**); 1:5 pysical mixtures (**C**); and SDs (**D**).

The thermal behavior of pure aprepitant, Soluplus^®^, physical mixtures and solid dispersions are shown in Figure 2. The pure aprepitant showed a melting endothermic peak at 253.2 °C. The endothermic peak for the drug disappeared in the solid dispersions indicating the absence of a melting endotherm of aprepitant. This showed that aprepitant was present in an amorphous state in the prepared solid dispersions.

### 2.3. X-ray Powder Diffraction (XRPD)

**Figure 3:**
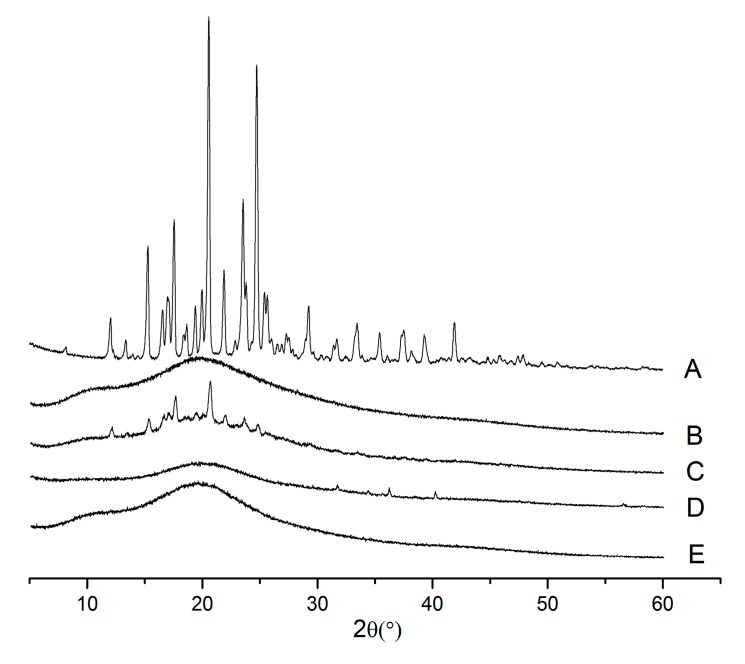
XRPD spectra of Aprepitant (**A**); Soluplus^®^ (**B**); 1:5 pysical mixtures (**C**); 1:1 SDs (**D**); 1:5 SDs (**E**).

The X-ray powder diffraction images of aprepitant, Soluplus^®^, physical mixtures and SDs are shown in Figure 3. Pure aprepitant exhibited sharp and intense peaks in the range of 5°–60° at 2θ angles, which suggested that aprepitant was present in crystalline form. All the major characteristic crystalline peaks of aprepitant were clearly observed in the PMs diffractograms. However, for the SDs (1:5), the discriminatory peaks of aprepitant were clearly absent, which indicated that aprepitant might exist in an amorphous state, which is consistent with the DSC results.

### 2.4. Scanning Electron Microscopy (SEM)

**Figure 4:**
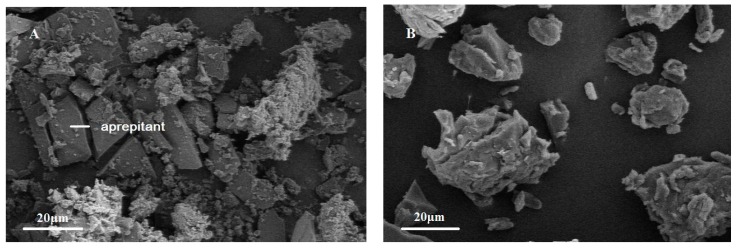
Scanning electron microscopy (SEM) photomicrographs of PMs (**A**) and SDs (**B**).

The morphology of the PMs and SDs was examined using SEM and the photographs are shown in Figure 4. Aprepitant powder appeared as plate-like crystals with smooth surfaces. However, the SDs appeared as irregular-shaped particles. In contrast, there was no crystal structure of aprepitant in SDs, indicating the transformation of aprepitant into an amorphous form, which agrees with the results of the DSC experiment.

### 2.5. Fourier Transform Infrared Spectroscopy (FTIR)

**Figure 5:**
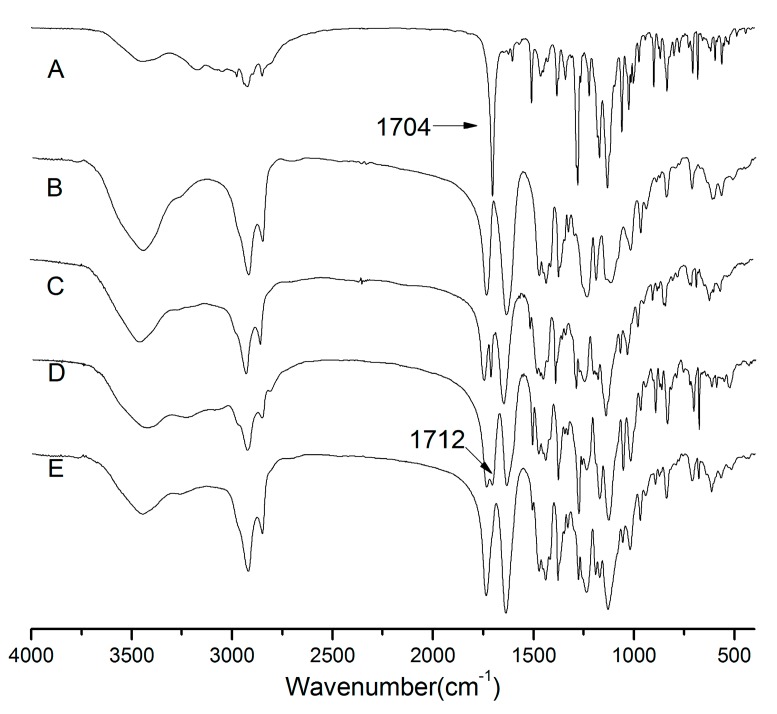
FTIR spectra of Aprepitant (**A**); Soluplus^®^ (**B**); 1:5 pysical mixtures (**C**); 1:1 SDs (**D**); 1:5 SDs (**E**).

Infrared spectroscopy has been widely used to investigate possible drug–polymer interactions in solid dispersion systems. From the chemical structures, hydrogen bonding could be expected between the hydroxyl groups of Soluplus^®^ and the carbonyl function of aprepitant [24]. In order to evaluate any possible solid–solid interactions between the drug and carriers, FTIR spectra of aprepitant, physical mixtures, and SDs were recorded and the results are shown in Figure 5.

The FTIR spectra obtained for aprepitant presented characteristic peak C=O stretching at 1704 cm^−1^, C-F stretching at 1132 cm^−1^, C-H stretching over the range 1500–1600 cm^−1^ [10]. The Soluplus^®^ spectra showed a C=O peak at 1740 cm^−1^ and 1641 cm^−1^, and an OH peak at 3447 cm^−1^. The FTIR spectra of physical mixtures were similar to those of aprepitant and Soluplus^®^ individual spectra, which suggest that there was no chemical interaction between aprepitant and Soluplus^®^ in physical mixtures.

The spectrum of SDs (1:5) showed that a weak-OH stretching vibration peak was observed at 3447 cm^−1^ and the peak of the drug at 1704 cm^−1^ was absent, and only the C=O peak at 1740 cm^−1^ and 1641 cm^−1^ of the polymer was present. The spectra of the SDs (1:1) showed a shift of 1704 cm^−1^ (C=O) of the crystal drug to 1712 cm^−1^. This finding suggested that aprepitant interacted with Soluplus^®^, presumably by hydrogen bonding. It is important to mention that this kind interaction between drug and carrier is an additional benefit for the SDs, since they can increase the solid solubility of the drug into the hydrophilic carrier besides acting on inhibiting the crystallization of drug [25].

### 2.6. In Vivo Evaluation

**Figure 6:**
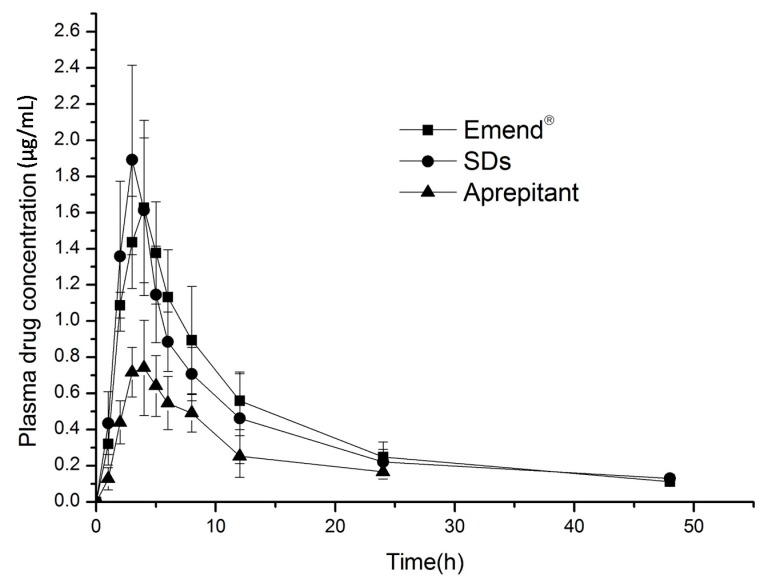
Plasma concentration-time profiles following oral administration of solid dispersions and Emend^®^ (*n* = 6).

The plasma drug concentration-time profiles and pharmacokinetics of aprepitant resulting from the SDs, Emend^®^ and aprepitant in rats are shown in Table 1 and Figure 6. The AUC from 0 to 48 h of the SDs and Emend^®^ was 19.10 ± 3.10 μg·h/mL and 20.51 ± 3.60 μg·h/mL, respectively, which yielded a relative bioavailability of 93.12%. For the aprepitant, the AUC_0–t_ and C_max_ were 7.97 ± 1.67 μg·h/mL and 0.82 ± 0.14 h. The AUC_0–t_ of the SDs was 2.4-fold that of the aprepitant. The T_max_ of the SDs (3 ± 0.63 h) was markedly shorter than that of Emend^®^ (4.33 ± 0.52 h). The difference in T_max_ could be attributed to their dissolution rates and solubility. The better solubility of the amorphous state of aprepitant indicated faster absorption. However, the better oral bioavailability of Emend^®^ could be explained by the following effects: firstly, the smaller drug particles with increased surface area and reduced diffusion layer thickness were absorbed more rapidly through the gastrointestinal wall [26]; secondly, the surfactants in the formulation would improve the dissolution and absorption of aprepitant in the gastrointestinal tract. The ingredients of Emend^®^ contain sucrose, microcrystalline cellulose, hydroxypropyl cellulose and solium lauryl sulfate [7].

**Table 1:** Pharmacokinetic parameters following oral administration of solid dispersions and Emend^®^ (*n* = 6).

Parameter	Emend^®^	Solid Dispersions	Aprepitant
AUC_0–48 h_ μg·h/mL	20.51 ± 3.60	19.10 ± 3.10 *	7.97 ± 1.67
AUC_0–∞_ μg·h/mL	22.21 ± 3.96	20.09 ± 3.93	9.42 ± 2.29
C_max_ μg/mL	1.71 ± 0.40	2.085 ± 0.30	0.82 ± 0.14
T_max_ h	4.33 ± 0.52	3 ± 0.63	3.67 ± 0.52

* Statistically significant compared with the Emend^®^ (*p* > 0.05).

### 2.7. Stability Study

**Figure 7:**
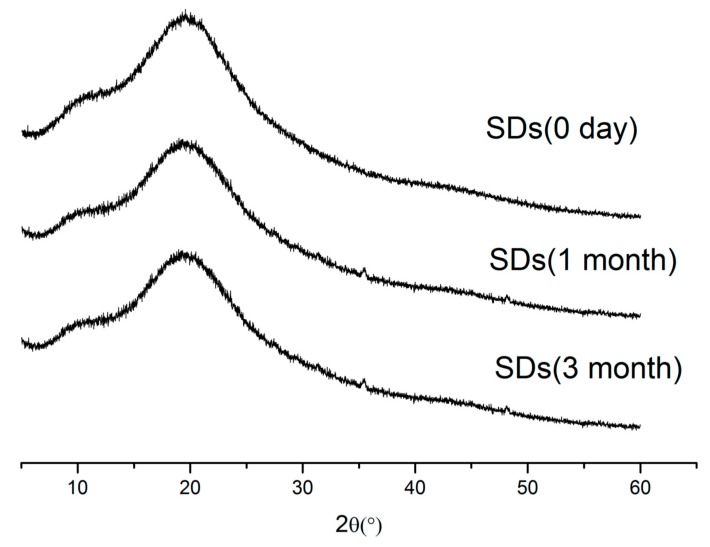
XRPD spectra for SDs stored 0 day, 1 month, 3 month.

Stability studies of the physical state of the SDs were performed using XRPD. The data obtained are shown in Figure 7. As can be seen from the XRPD data, the SDs remained amorphous for three months.

## 3. Experimental Section

### 3.1. Materials

Aprepitant with 99% purity was purchased from Wuhan Kailun Chemical & Advanced Materials Co., Ltd., Wuhan, China. Soluplus^®^ was supplied by BASF, Ludwigshafen, Germany. Emend^®^ was obtained commercially (Merck & Co., Inc., Summit, NJ, USA). Acetone (analytical grade), and acetonitrile (chromatographic grade) were purchased from Yuwang Industrial Co., Ltd., Jinan, China. Deionized water used in all the experiments, and all other chemicals were of analytical reagent grade.

### 3.2. Preparation of the Solid Dispersions

Solid dispersions of aprepitant with Soluplus^®^ were prepared using the solvent evaporation method. Aprepitant and Soluplus^®^ (drug to Soluplus^®^ weight ratios of 1:3, 1:4, 1:5, 1:6) were dissolved in 10 mL acetone. The solvent was removed by evaporation by heating under constant stirring. The solid dispersions was stored overnight in vacuum. After drying, the product was ground using a mortar and pestle and sieved through 50-mesh screen. Subsequently, samples were stored in a desiccator until required for further use.

### 3.3. Dissolution Studies

The dissolution studies were carried out according to the Chinese Pharmacopeia (2005 ED) Method II, and a paddle method using a ZRS-8L dissolution apparatus (Tianda Tianfa, Tianjin, China) was used. Solid dispersions equivalent to 25 mg pure aprepitant were placed in 250 mL PBS 6.6 containing 0.1% sodium dodecyl sulfate and stirred at 37 ± 0.5 °C and 50 rpm. At predetermined times, 0.6 mL samples were withdraw and replaced with more medium to maintain a constant volume. The withdrawn samples were passed through a 0.45 µm membrane filter and the mount of drug dissolved at different times was determined using a validated HPLC method.

### 3.4. Differentinal Scanning Calorimetry (DSC)

Thermograms of aprepitant, physical mixtures(PMs) and SDs were obtained using a differential scanning calorimeter (DSC-1, METTLER, Zurich, Switzerland).Samples of about 3 mg were approximately weighed and analyzed in pierced Al crucibles over the range from 30–300 °C, at a heating rate of 10 °C·min^−1^ in a nitrogen atmosphere.

### 3.5. Analysis of X-ray Powder Diffraction (XRPD)

XRPD analysis were performed in a universal diffractometer (X’Pert PRO, PA-Nalytical, Almelo, Holland) with Cu Kα monochromatic radiation(λ = 1.540598 Å). The tubeanode X-ray was operated at 40 kV and 40 mA. The samples were placed in an aluminum sample port, and the X-ray deflection was measured from 5° to 60° with a step size of 0.01670° 2θ.

### 3.6. Scanning Electron Microscopy (SEM)

The morphology of PMs and SDs were observed using a scanning electron microscope (S-3400, Hitachi, Japan). Samples were coated with gold and palladium using a vacuum evaporator and then examined at an accelerating voltage of 10 kV.

### 3.7. Fourier Transform Infrared Spectroscocopy (FTIR)

FTIR measurements were carried out using an infrared spectrophotometer, (EQUINOX55, Bruker, Karlsruhe, Germany) at room temperature. Samples of aprepitant, Soluplus^®^, PMs, and SDs were ground and mixed thoroughly with potassium bromide before measurements were carried out. The scanning range was 400–4000 cm^−1^ and the resolution was 1 cm^−1^.

### 3.8. In Vivo Experiments

#### 3.8.1. Animal Experiments

Male Sprague-Dawley rats (200–240 g) were used and randomly divided into three groups (*n* = 6). The rats were fasted for 12 h prior to the experiments but granted free access to water. Three formulations (SDs, Emend^®^ (equivalent to 25 mg/kg aprepitant) and aprepitant) were dispersed in 1 mL distilled water with 0.5% CMS-Na and given orally to the three groups of rats. Blood samples (0.3 mL) were collected from the postorbital venous plexus at 1, 2, 3, 4, 5, 6, 8, 12, 24 and 48 h postdosing. The collected blood samples were centrifuged and the plasma was stored at −20 °C until required for HPLC analysis.

#### 3.8.2. Plasma Processing and Analysis

The plasma samples (100 μL) were mixed with 10 μL internal standard solution (gliclazide, 10 μg/mL), and vortexed for 1 min. Then, the samples were made alkaline with 100 μL 0.1 M NaOH followed by further vortexing for 1 min. After this, 2.5 mL extraction solvent (ethyl acetate) was added and vortexed again for 3 min. After centrifugation at 4000 rpm for 5 min, the organic phase was transferred to another tube and evaporated to dryness at 40 °C under a gentle stream of nitrogen. The dried residue was redissolved in 100 μL mobile phase and a 20 μL sample was injected into the HPLC system.

All samples were analyzed by HPLC (LC-10A VP pump equipped with a SPD-10A VP UV detector, Shimadu, Japan). The separation was carried out on a Phenomenex C8 column (150 mm × 4.6 mm, 5 μm) and the mobile phase was composed of a mixture of 0.05% phosphate and acetonitrile (50:50, *v*/*v*). The flow rate was 1.0 mL/min, and the UV detector was set at 220 nm. The column temperature was maintained at 30 °C. All pharmacokinetic parameters were calculated by non-compartmental analysis using a DAS package (Version 2.1, Mathematical Pharmacology Professional Committee, Beijing, China). The data were presented as mean ± SD, and the Student’s *t*-test was used to analyze differences.

#### 3.8.3. Stability Studies

The prepared SDs were sealed with aluminum foil and stored for three months at 40 °C and 60% relative humidity in an artificial climate box. XRPD analysis was conducted to monitor any potential crystallization of the solid dispersions.

## 4. Conclusions

In this study, aprepitant solid dispersions with Soluplus^®^ were successfully prepared and characterized. The dissolution rate of aprepitant was significantly increased by solid dispersion technology. FTIR spectrometry suggested the presence of intermolecular hydrogen bonds between aprepitant and Soluplus^®^, DSC, SEM and XRPD analysis indicated the stability of the amorphous state of the drug in SDs. Furthermore, pharmacokinetic studies in rats indicated that the drug absorption was comparable with that of Emend^®^.
